# Quantitative analysis of stem-palaeognath flight capabilities sheds light on ratite dispersal and flight loss

**DOI:** 10.1098/rsbl.2025.0320

**Published:** 2025-09-17

**Authors:** Klara Widrig, Fabio Alfieri, Pei-Chen Kuo, Helen James, Daniel J. Field

**Affiliations:** ^1^ Department of Vertebrate Zoology, Smithsonian National Museum of Natural History, Washington, DC, USA; ^2^ Department of Earth Sciences, University of Cambridge, Cambridge, UK; ^3^ Institute of Ecology and Evolution, Universitat Bern, Bern, BE, Switzerland; ^4^ Negaunee Integrative Research Center, Field Museum of Natural History, Chicago, IL, USA; ^5^ Museum of Zoology, University of Cambridge, Cambridge, United Kingdom

**Keywords:** Aves, Palaeognathae, Lithornithidae, biogeography, functional morphology, geometric morphometrics

## Abstract

Lithornithids are an assemblage of Palaeogene fossil birds thought to represent stem-group members of Palaeognathae. Among extant palaeognaths, which include flightless ratites such as ostriches, only tinamous can fly, though only in anaerobic bursts. Despite their limited dispersal capabilities, the phylogenetic interrelationships and geographic distributions of palaeognaths imply that their early relatives were capable of long-distance dispersal, although quantitative skeletal evidence has not been applied to this question. We investigate the flight capabilities and ecology of the Palaeogene lithornithid *Lithornis promiscuus* using a three-dimensional geometric morphometric dataset spanning the avian crown group. Our models reject the hypothesis that *Lithornis* would have relied on tinamou-like burst flight, and show that its sternum morphology is consistent with a range of aerobic, flapping flight styles—closely resembling those of many extant birds exhibiting pronounced dispersal capabilities. Our results are consistent with inferences from lithornithid wing shape, supporting the hypothesis that at least some stem palaeognaths were capable of long-distance flight, helping to clarify the origins of the transoceanic distributions of extant flightless ratites.

## 1. Introduction

The historical biogeography of flightless and poorly flighted birds has long attracted research attention. Indeed, the origins of the present-day geographic distributions of palaeognaths, the sister group to all other extant birds, have been the subject of much debate. Palaeognathae includes the flightless ratites, which are represented by two species of ostrich in Africa (the common ostrich, *Struthio camelus*, persisted until recently in Asia Minor) [1], two rhea species in South America, the monotypic emu and three cassowary species in Australia and New Guinea, and up to five kiwi species in New Zealand [2]. In addition to ratites, Palaeognathae also encompasses 46 species of tinamou in Central and South America [2]. Tinamous are the only extant palaeognaths capable of flight, albeit only over short bursts to escape from danger, traverse barriers (e.g. rivers) or roost in trees [3]. Recently extinct ratite species include up to four elephant birds in Madagascar [4,5] and nine moa in New Zealand [6]. Collectively, five Gondwanan landmasses separated by oceanic barriers hosted ratites in the recent past, with ostriches additionally occurring in Asia [1,7].

Ratite flightlessness, combined with their widespread distributions, has posed a perennial historical biogeographic challenge. Gondwanan vicariance was long assumed to have driven phylogenetic divergences among crown palaeognaths [8–10]; however, temporal estimates for the break up of Gondwana [11,12] combined with refined phylogenetic divergence times [13–17] firmly reject this hypothesis, demonstrating that crown palaeognaths diversified far too recently for their distributions to be explicable by continental vicariance. Moreover, the realization that ratites are not monophyletic, but paraphyletic with respect to tinamous [13–30], casts doubt on the longstanding assumption that the last common ancestor of ratites was flightless, suggesting the possibility of pronounced dispersal capabilities early in palaeognath evolutionary history.

Hosner *et al.* [31] found that the poorly dispersive partridges *Margaroperdix* of Madagascar and *Anurophasis* of New Guinea are phylogenetically nested within *Coturnix* quails, which have a greater capacity for long-distance flight. The authors proposed an evolutionary cycle in which members of a clade first acquire dispersal capabilities, disperse to isolated landmasses, then give rise to non-dispersive descendants. In a scenario perhaps applicable to palaeognaths, subsequent extinction of the dispersive ancestral taxa could give rise to confounding biogeographic patterns.

The earliest fossil evidence of total-group Palaeognathae is provided by Lithornithidae, an apparently volant group from the Palaeogene of Europe and North America [32]. Phylogenetic analyses employing molecular scaffolds recover lithornithids as stem-group palaeognaths [33,34]. Whereas burst-flying tinamous are characterized by short wings, high wing loadings and massive pectoral muscles [35–37], the lithornithid flight apparatus is quite different. The sterna of tinamous and other burst fliers tend to be caudally elongate and deeply notched, while the sternum of lithornithids is comparatively short with an unnotched posterior margin, suggesting that lithornithids lacked the hypertrophied pectoral muscles associated with burst flight [32]. Additionally, the lithornithid humerus is proportionally longer than that of tinamous, with the insertion for the pectoralis musculature situated further distally, which may reflect a functional trade-off, allowing for greater leverage at the cost of wingbeat speed [32]. Furthermore, investigation of a specimen of the lithornithid *Calciavis grandei* [38] (*Lithornis* cf. *grandei* following Mayr & Kitchener [39]) preserving carbonized feather traces suggested that it may have predominantly engaged in continuous flapping flight based on reconstructed wingspan and wing surface area, congruent with the potential for sustained flight over long distances [38].

Despite these inferences into dispersal potential from *Calciavis*, quantitative assessments of the musculoskeletal flight apparatus have not been undertaken to assess whether lithornithids were capable of long-distance flight rather than the burst flight used by their extant relatives. Here, we investigate the lithornithid flight apparatus to independently assess flight capacity, drawing on the exceptional undistorted, three-dimensionally preserved *Lithornis promiscuus* (USNM 336535) from the late Palaeocene–earliest Eocene Willwood Formation of Wyoming. We focus on the sternum, which serves as the origin of the largest flight muscles and reliably facilitates inferences of flight style in extant [40] and extinct birds [41].

## 2. Methods

### (a) Scanning and geometric morphometric analysis

The *Lithornis promiscuus* (USNM 336535) sternum (electronic supplementary material, figure S1) was scanned on a GE phoenix v∣tome∣x m microCT scanner at the Smithsonian National Museum of Natural History (USNM) (90 kV, 280 μA, 3000 frames, voxel size 42.5 μm). Scans were processed in VGSTUDIO MAX v. 3.4 (Volume Graphics, Heidelberg, Germany) and exported as a .stl. Landmarks and semilandmarks were placed in Avizo 2019.3 (Thermo Fisher Scientific). We used the landmarking scheme and dataset of Bjarnason & Benson [42], which contains 149 taxa and samples most neornithine subclades. The scheme contains 12 point landmarks and eight semilandmark series, for a total of 67 landmarks per sternum. We removed the Abyssinian ground hornbill *Bucorvus abyssinicus*, as the landmarking scheme does not account for the atypical shape of this sternum due to its mediolaterally expanded carinal apex. We added taxa from the University of Cambridge Museum of Zoology (UMZC) and Field Museum of Natural History (FMNH) to increase sample sizes within each flight category from Kotnour *et al.* [43]. We assigned birds to seven categories (table 1) based upon information from the *Handbook of birds of the world* [44], the Internet Bird Collection [44] and the Macaulay Library [45], as well as published flight style assignments [40,43].

**Table 1.** Flight style categories. Definitions of all categories follow Kotnour *et al*. (2022) except for burst flight (added for this study) and powered swimming (amended to include volant wing-propelled divers).

flight style category	definition
continuous flapping (CF)	wing flapping with no or rare periods of soaring or gliding
soaring (SO)	primarily soaring with very little wing flapping
flap-gliding (FG)	regular periods of flapping and gliding with wings outstretched
intermittent bounding (IB)	regular periods of flapping and gliding with wings held against the body
hovering (H)	stationary hovering
wing-propelled swimming (PS)	regular use of wings to generate thrust underwater
burst flight (BF)	anaerobic flight with rapid continuous wingbeats over a short distance.

We performed a generalized Procrustes analysis using the ‘gpagen’ function in the R package *geomorph* v. 4.0.5 [46,47]. We used the minimum bending energy sliding method for semilandmarks [48,49]. We performed principal components analysis (PCA) on Procrustes coordinates using the ‘gm.prcomp’ function in *geomorph*, and warped the sternum closest to the centroid of principal component space (*Ptilonorhynchus violaceus*) to represent positive and negative extremes for the first three axes. Following Kuo *et al.* [50], we used thin-plate spline deformation [51] using the functions ‘tps3d’ from the package *Morpho* v. 2.10 [52] and ‘shape.predictor’ from *geomorph* to produce the warped sterna, and plotted landmark constellations coloured according to the amount of deformation using the ‘hot.dots’ function [53]. We created convex hulls for each flight style using the ‘geom_polygon’ function in *ggplot2* v. 3.4.1 [54]. To determine the sterna exhibiting the least and greatest geometric similarity to *Lithornis*, we calculated pairwise Euclidean distances between *Lithornis* and all other taxa in multivariate shape space. We additionally performed a phylogenetically informed PCA using the ‘phyl.pca’ function in *phytools* v. 1.5.1 [55–57] and extracted Euclidean distances similarly.

### (b) Multivariate statistical analysis of shape data

We performed multivariate phylogenetic generalized least squares (PGLS) regressions [58] using the function ‘procD.pgls’ in *geomorph* to investigate relationships among sternum shape, sternum size, body mass, flight style and phylogenetic structure, as well as between sternum size and body mass (electronic supplementary material, table S2). We derived our phylogenetic backbone by pruning the densely sampled composite tree of Cooney *et al.* [59] using the ‘drop.tip’ function in the R package *ape* v. 5.7 [60]. As not all taxa within the Bjarnason & Benson [42] dataset are represented in the Cooney *et al.* [59] tree, we added missing taxa and branch lengths in Mesquite 3.61 [61]. The branch length for *Lithornis promiscuus* was estimated based on the midpoint of its stratigraphic range of 59.2 to 56 Ma [32,62,63]. The phylogenetic position of *Lithornis* follows the molecular scaffold tree of Nesbitt & Clarke [33]. We obtained body mass data from Lowi-Merri *et al.* [40], Kotnour *et al.* [43], *Birds of the world* [64] and AVONET [65]. These data were log10 transformed, and log-transformed centroid size was used to approximate the size of each sternum [66].

Because the ‘procD.pgls’ function assumes a Brownian motion model [47], we followed the method of Fabbri *et al.* [67] and Kuo *et al* [50] to estimate the value of Pagel’s lambda to rescale our phylogenies for each analysis such that the phylogenetic–variation–covariation matrix is compatible with the assumption of Brownian motion. We ran each PGLS analysis once, then used the residuals to calculate Pagel’s lambda using the ‘phylosig’ function. After using this value of lambda to rescale the phylogeny using the ‘rescale’ function in the R package *geiger* v. 2.0.10 [68], we re-ran the PGLS analysis.

Differences in sternal morphology among flight categories were further tested through an alternative approach. We first fitted a multivariate phylogenetic linear model using the ‘mvgls’ function (mvMORPH package v. 1.1.9 [69]). We applied this function to the first five principal components of the geometric dataset against our locomotor categories, then performed a type II phylogenetic multivariate analysis of variance (MANOVA) on the fitted data using the ‘manova.gls’ function from mvMORPH to determine whether significant differences existed between flight style categories under the fitted model. Although this is partially redundant with our ‘shape ~ flight style’ PGLS analysis, it was required for subsequent classification approaches. Significance was assessed using a Pillai statistic with 1000 permutations. After detecting significant differences through MANOVA, we tested which significant differences existed between flight styles in every possible pairwise comparison using ‘pairwise.ghl’ from the mvMORPH package, again using a Pillai statistic and 1000 permutations, with a Bonferroni adjustment (electronic supplementary materials, tables S4 and S5).

We applied a phylogenetic discriminant function analysis (pDFA) to predict the flight style of *Lithornis* following the methods of Monclús-Gonzalo *et al.* [70]. Using the fitted MVGLS model derived using the ‘mvgls’ function in mvMORPH as an argument of the function ‘mvgls.dfa’ from mvMORPH, we created a pDFA model. To assess the model’s performance, we ran it on a training dataset containing only extant taxa (those with known flight styles). We assessed the misclassification rate via leave-one-out cross-validation (LOOCV), in which each extant species in the training dataset was removed one at a time, and the flight style of the omitted species was predicted from the model trained on those remaining. Using the tested pDFA model, the flight style of *Lithornis* was estimated using the ‘predict’ function, assuming equal priors. Again, we applied LOOCV subsampling to estimate the rate of assignment of *Lithornis* to each flight style. The entire protocol was performed three times, pooling flight style categories to determine whether applying more inclusive categories affected the misclassification rate. Thus, the protocol (i.e. mvgls + MANOVA + pairwise comparisons + pDFA + locomotor prediction) was performed on a dataset using all seven flight style categories; one with continuous flapping and flap gliding pooled into a generalized flapping category; and one in which all birds were categorized as either aerobic (non-burst fliers) or burst fliers.

## 3. Results

### (a) Geometric morphometrics

**Figure 1.**
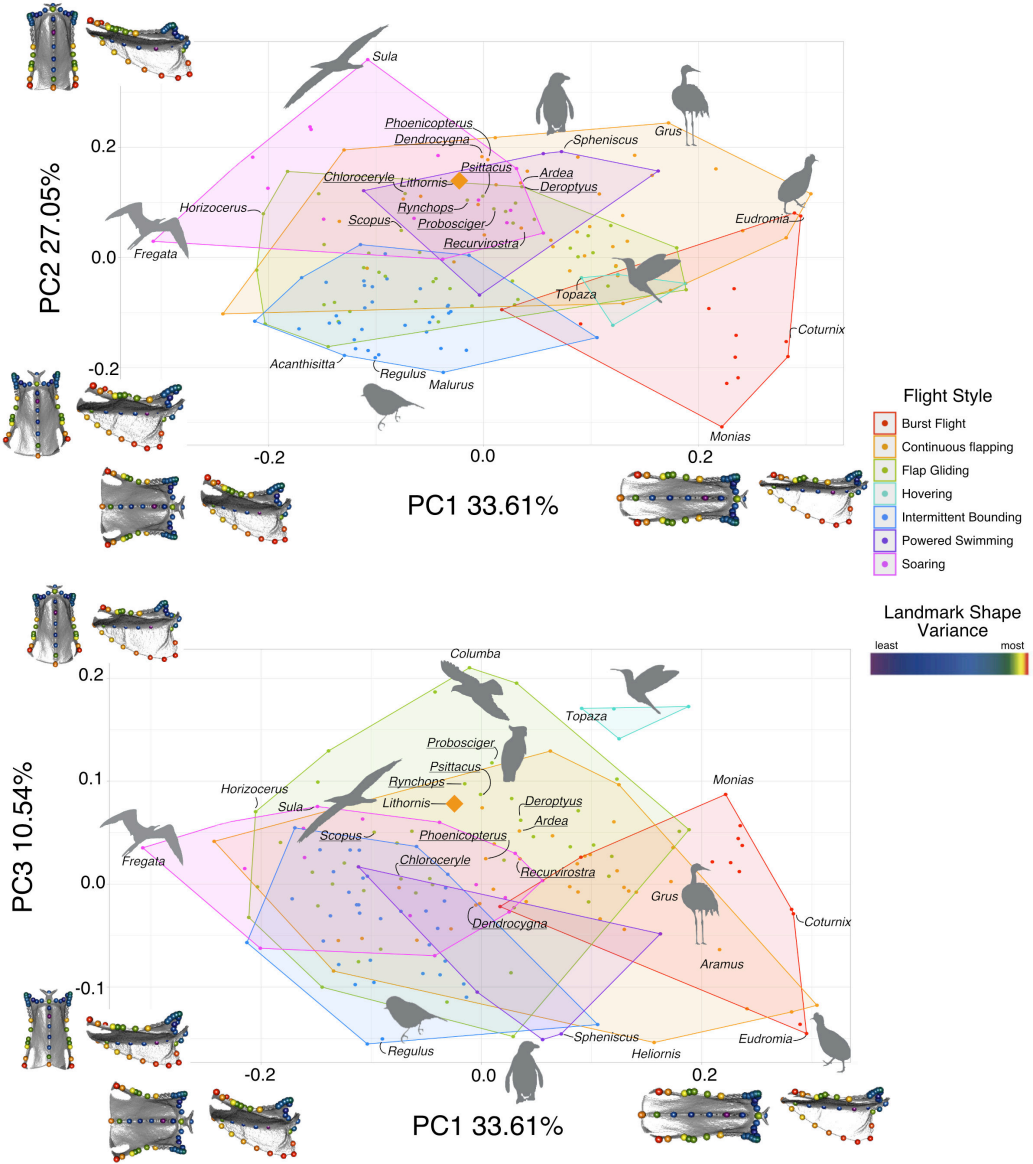
Morphospace of the first three axes of sternum shape variation. Convex hulls represent the approximate area of morphospace occupied by each flight style. The 10 most similar taxa to *Lithornis*, as well as notable examples of each flight style, are labelled. The 10 most similar taxa are underlined. Sternum models in dorsal (left) and left lateral (right) views represent the 0.1 and 0.9 quantiles of shape variation along each axis. Landmarks on the sterna are coloured according to log10-transformed per-landmark Procrustes variance. Silhouettes © the authors.

The first five principal components account for 83.36% of shape variation (PC1: 33.61%, PC2: 27.05%, PC3: 10.54%, PC4: 7.27%, PC5: 4.91%; figure 1). PC1 is associated with the degree of caudal elongation, with longer sterna occupying the positive side of the PC1 axis and shorter sterna occupying the negative side. PC2 captures the degree of projection of the sternal keel, with keels that project further cranially occupying positive PC2 values. PC3 is correlated with keel depth, with deeper keels associated with positive PC3 scores. These results agree with Lowi-Merri *et al.* [40].

**Figure 2.**
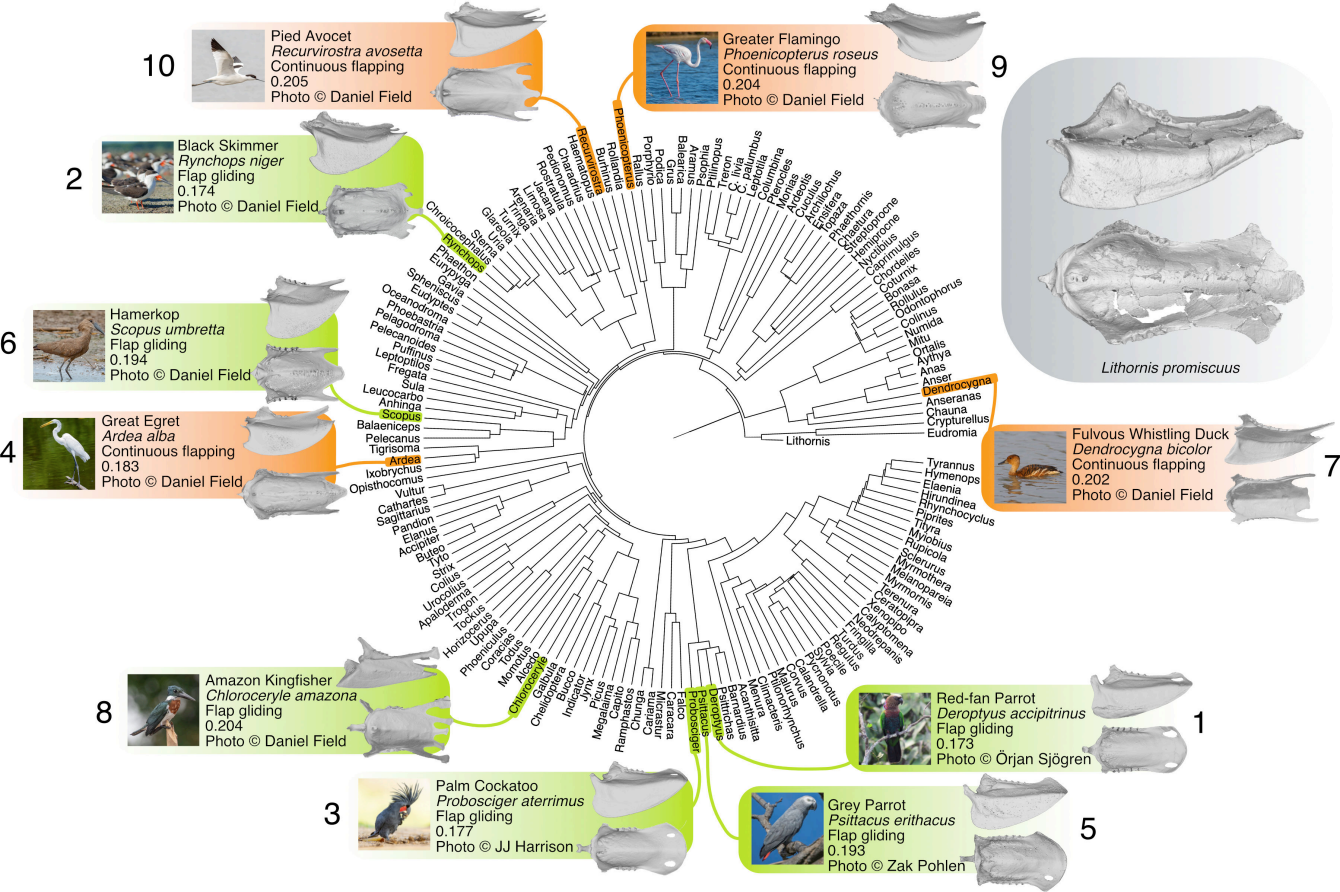
Time-calibrated phylogenetic relationships of taxa in our geometric morphometric analysis highlighting the top 10 most similar sterna to *Lithornis promiscuus* by Euclidean distance. Common names, Latin names, primary flight style and Euclidean distance from *L. promiscuus* are listed for each species, and left lateral (top) and dorsal (bottom) views of each sternum are provided. Inset depicts the *L. promiscuus* sternum in left lateral (top) and dorsal (bottom) views. Continuous flapping birds are highlighted in orange, and flap-gliders are in green. Taxa are numbered from most to least similar to *L. promiscuus*.

**Figure 3.**
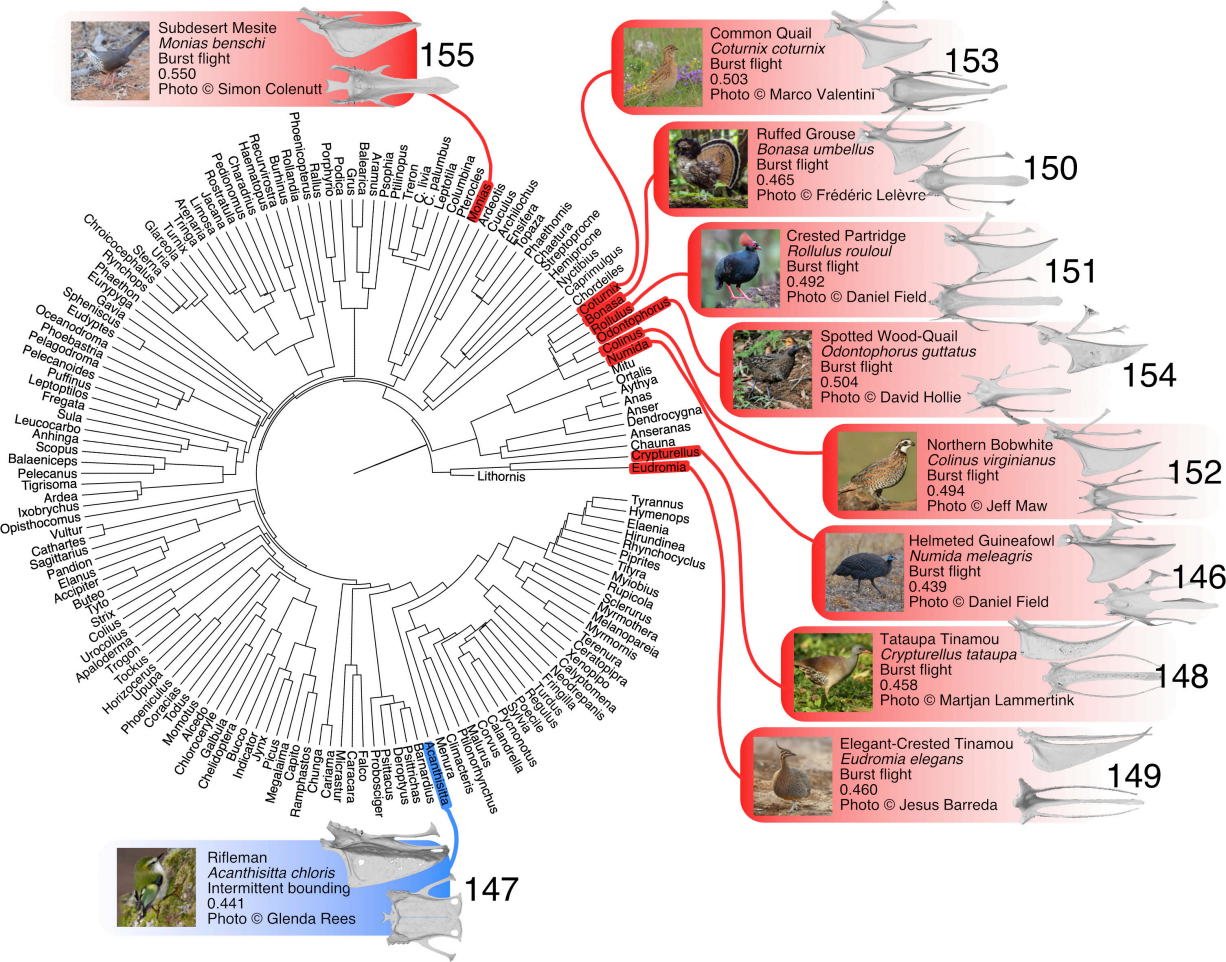
Time-calibrated phylogenetic relationships of taxa in our geometric morphometric analysis highlighting the top 10 most dissimilar sterna to *Lithornis promiscuus* by Euclidean distance. Common names, Latin names, primary flight style and Euclidean distance from *L. promiscuus* are listed for each species, and left lateral (top) and dorsal (bottom) views of each sternum are provided. Burst fliers are highlighted in red, and intermittent bounders in blue. Taxa are numbered from most to least similar to *L. promiscuus*.

Despite some overlap, burst fliers occupy a distinct region of PC1–PC2 morphospace, characterized by elongation of the sternum (positive PC1 values) and a less cranially projected apex of the keel (mostly negative PC2 values). Likewise, soaring birds are relatively distinct, showing the opposite trend, i.e. mostly negative PC1 and positive PC2 scores. Intermittent bounding birds show mainly negative PC1 and PC2 values, though there is broad overlap with continuous flapping, flap-gliding and soaring birds. Wing-propelled swimmers and hovering birds are differentiated in PC1–PC3 morphospace, with swimmers exhibiting shallow keels and hoverers exhibiting especially deep keels. Continuous flapping and flap-gliding birds occupy an overlapping area of morphospace spanning nearly the entire range of PC1 and PC3 values, while tending towards intermediate values on PC2. *Lithornis promiscuus* falls within the region of overlap between soaring, wing-propelled swimming, continuous flapping and flap-gliding in PC1–PC2 morphospace, and in the region of overlap between continuous flapping and flap-gliding in PC1–PC3 morphospace. The 10 taxa with the most similar sterna to *Lithornis* based on Euclidean distance fall into either the continuous flapping or flap-gliding flight style categories (figure 2), while the least similar taxa are almost all burst fliers (figure 3). The phyloPCA identified similar patterns to the traditional PCA (electronic supplementary materials, figure S2, tables S8 and S9).

### (b) Multivariate statistics

Our PGLS analyses (electronic supplementary materials, tables S2 and S3) indicate that sternum size is strongly correlated with body mass (*R*^2^ = 0.91223, *Z* = 12.169, *p* = 1 × 10^−04^), as found in previous investigations [40,41]. Shape and flight style show a significant association, though with a low proportion of variance explained (*R*^2^ = 0.12723, *Z* = 6.2448, *p* = 1 × 10^−04^). We also found a strong correlation between sternum shape and phylogenetic group (*R*^2^ = 0.69108, *Z* = 8.2025, *p* = 1 × 10^−04^), indicating a non-negligible phylogenetic signal. When this analysis was repeated on a dataset excluding burst fliers, phylogenetic signal dropped by a large amount (*R*^2^ = 0.29963, *Z* = 6.2448, *p* = 1 × 10^−04^). Significant associations but with very low proportions of variance explained were found between shape and centroid size (*R*^2^ = 0.0705, *Z* = 5.5709, *p* = 1 × 10^−04^), and shape and body mass (*R*^2^ = 0.05982, *Z* = 5.0896, *p* = 1 × 10^−04^), suggesting very weak allometric effects.

The ability of our pDFA model to categorize flight styles of extant birds was relatively poor for the analyses with seven and six categories, consistent with the low *R*^2^ from the ‘shape ~ flight style’ PGLS, with correct classification rates of 52 and 55%, respectively. Thus, we consider categorizations derived from these models to be unreliable. The pDFA model fared better in the binary categorization of birds as burst fliers or non-burst (aerobic) fliers, with a correct classification rate of 88%. *Lithornis* was predicted to be an aerobic, non-burst flier in all resampled runs.

## 4. Discussion

We interpret that the flight style of *Lithornis promiscuus* may have been characterized by continuous flapping and/or mixed flapping and gliding, rejecting the interpretation that it was a burst flier like extant flying palaeognaths. The considerable overlap in morphospace between soaring, flap-gliding and continuous flapping may reflect the non-independence of these flight style categories, as some birds facultatively switch among them, and may be better understood as unspecialized fliers. While necessary for the analyses undertaken here (e.g. pDFA), applying strict flight style categories has drawbacks, as subjective assessments may be introduced when defining a species’ most frequently used flight style [71]. For example, the Siberian crane *Grus leucogeranus* is classified here as a continuous flapping bird, though cranes also employ soaring and flap-gliding flight [72]. The position of *Lithornis* within the region of overlapping morphospace occupied by non-mutually exclusive flight categories suggests it may also have been a generalist aerobic flier, perhaps primarily employing continuous flapping (like many taxa plotting within the same region of morphospace) but also capable of other aerobic flight styles.

The relatively low proportion of shape variance explained by flight style (*R*^2^ = 0.13, electronic supplementary material, table S2) is consistent with the observation that sternum shape is only a reliable proxy for separating burst and non-burst fliers, as shown by our pDFA results. Due to co-variation between shape and phylogenetic groups with a higher proportion of explained variance (*R*^2^ = 0.69, electronic supplementary material, table S2), we cannot exclude that some patterns in our morphospaces reflect phylogenetic signal. Nonetheless, those taxa exhibiting geometries similar to *Lithornis* represent a phylogenetically diverse assemblage (figure 2, electronic supplementary material, table S6), suggesting that while phylogenetic signal may influence the distribution of taxa within sternum morphospace, its effects on the functional patterns of interest here (i.e. those relative to *Lithornis*) are minor, a conclusion also supported by our phyloPCA results (electronic supplementary materials, figure S2, tables S8 and S9). Indeed, *Lithornis* is extremely dissimilar to its closest relatives (Tinamidae; figure 3, electronic supplementary material, table S6). In addition to tinamids, the taxa with the least geometrically similar sterna to *Lithornis* are predominantly burst-flying landfowl (Galliformes; figure 3), which exhibit striking ecomorphological convergence with tinamids. The lower phylogenetic signal obtained when burst fliers are excluded likely reflects the fact that all galliforms in the dataset employ burst flight (electronic supplementary material, table S3).

Many of the 10 least similar taxa are incapable of traversing oceanic barriers. As examples, tinamids do not naturally occur on oceanic islands [2], and the crested partridge is only found on islands that were connected to mainland Asia during the Pleistocene [73]. Mesites are endemic to Madagascar and are extremely reluctant fliers [2]. By contrast, supporting the interpretation that lithornithids were strong fliers with potential for range expansion, several species out of the 10 most geometrically similar are capable of long-distance dispersal (figure 2; electronic supplementary material, table S6). The great egret (electronic supplementary material, figure S3) is known to wander extensively, exhibiting one of the most cosmopolitan distributions of any living bird [74], while cases of transoceanic vagrancy are known for other taxa with similar sternum geometries (e.g. the greater flamingo [75]).

These insights may help clarify the mechanisms by which early palaeognaths achieved wide geographic distributions. Several ardeids, represented here by the great egret (electronic supplementary material, figure S2), have undergone dramatic natural range expansions, including cattle egrets (electronic supplementary material, figure S2), which comprise one of the most dramatic illustrations of avian dispersal and range expansion in modern times [76–80]. These remarkable range expansions have been attributed to strong dispersive behaviour in juveniles [79,81], strong flying potential in Ardeidae and the emergence of suitable habitat due to expanded livestock grazing around the world that allowed errant birds to thrive [82]. These examples, involving taxa with geometrically similar sternal morphologies to *Lithornis*, underscore the plausibility of long-distance dispersal of flying stem and/or early crown palaeognaths. Our findings also corroborate the conclusion that burst-flying adaptations should not be assumed to be ancestral for palaeognaths or for crown birds in general despite a tendency to optimize as such in extant-only datasets [36,83], reaffirming the value of incorporating fossil data into ancestral state reconstructions of extant clades [84–87].

## 5. Conclusions

The cycle of vagility [31] proposes that many poorly dispersive taxa occupying isolated landmasses may be descendants of vagile ancestors. This model, developed to explain the historical biogeography of island-dwelling galliforms, may apply equally to the colonization of disparate landmasses by stem group representatives of the major palaeognath subclades. Our results [88], congruent with earlier investigations, are compatible with long-distance dispersive capabilities in lithornithids. Dispersal events among the flighted ancestors of extant ratite lineages thus plausibly underpin the distinctive geographic distributions of palaeognaths, which must have undergone numerous independent transitions to flightlessness and gigantism [13–30,89]. While the details of palaeognath historical biogeography may remain elusive until clear evidence of flighted stem-group representatives of the major ratite subclades emerges, the present study advances our understanding of locomotor ecology in early palaeognaths, the sister group to all other extant birds.

## Data Availability

All materials needed to reproduce the results of this study are available here: [88]. A description of all files in this dataset is available in the README.txt file at the above link. Supplementary material is available online [90].
